# The Outcome of Fatherhood in Patients With Philadelphia-Negative Myeloproliferative Neoplasms: A Single-Institution Experience

**DOI:** 10.7759/cureus.25953

**Published:** 2022-06-15

**Authors:** Elrazi A Ali, Mohammad Abu-Tineh, Waail Rozi, Bashir Ali, Anas Babiker, Yousef Hailan, Qusai Al-Maharmeh, Zakaria Maat, Abdellatif Ismail, Mohamed A Yassin

**Affiliations:** 1 Internal Medicine, Hamad Medical Corporation, Doha, QAT; 2 Medical Oncology, Hematology, and Bone Marrow Transplant (BMT) Section, National Center for Cancer Care and Research, Hamad Medical Corporation, Doha, QAT; 3 Hematology and Oncology, Hamad General Hospital, Doha, QAT

**Keywords:** primary myelofibrosis, essential thrombocythemia, polycythemia vera, myeloproliferative neoplasm, male fertility, fatherhood

## Abstract

Background

Fertility is a highly complex subject; it involves more than one individual and has profound psychological and economic implications. Moreover, it is affected by several factors, including age, significant systemic illness in either partner, exposure to environmental toxins, medications, or radiation. In patients with malignancy, fertility is more complicated. Patients with a malignancy might have reduced fertility due to the disease, medication, and radiation. Besides the reduced fertility, there are more concerns regarding the subsequent effect of cancer treatment on their offspring and the possibility of having healthy children. There were many studies regarding fertility in patients with cancer; however, in male patients with Philadelphia-negative myeloproliferative neoplasms (MPNs), there are very limited data.

Objectives

In this study, we aim to see the outcome of fatherhood in male patients with Philadelphia-negative myeloproliferative neoplasms (MPNs), polycythemia vera (PV), essential thrombocythemia (ET), and primary myelofibrosis (PMF) whether on treatment or not.

Methods

A retrospective mixed-design study of male patients with Philadelphia-negative MPN was followed up in our institute (National Center for Cancer Care and Research (NCCCR)), Doha, Qatar, between January 1, 2008, and January 1, 2020. Patients were interviewed regarding fertility-related information. All included patients had a confirmed diagnosis of Philadelphia-negative MPN according to World Health Organization (WHO) 2008 or WHO 2016 criteria for MPN, aged more than 18 years old.

Results

A total of 124 male patients were interviewed, and only 20 patients met the inclusion criteria. The majority of the patients were lost to follow-up or could not be contacted, and 28.8% of the excluded patients had their families completed by the time of diagnosis. The treatment received included hydroxycarbamide (n=8), pegylated interferon 2 alpha (n=10), ruxolitinib (n=1), and phlebotomy (n=1). The mean duration of exposure to treatment before pregnancy was 4.7 years. The mode of delivery was normal vaginal delivery in 71.4% of the pregnancies. The total number of offspring was 30, and the total number of conceptions was 30.

Conclusion

Our data showed that most Philadelphia-negative MPN male patients on treatment had their offspring born normally with no serious complications, congenital anomalies, or reports of MPN-related cancers. Patients’ concerns regarding fertility should be addressed well to ensure a better quality of life.

## Introduction

Myeloproliferative neoplasms (MPNs) are a group of disorders characterized by terminal myeloid cell expansion in the peripheral blood [1]. They can be classified into Philadelphia-positive disorders, chronic myeloid leukemia (CML), and Philadelphia-negative disorders. Philadelphia-negative MPN includes polycythemia vera (PV), essential thrombocythemia (ET), primary myelofibrosis (PMF), and prefibrotic primary myelofibrosis (PMF). They are diagnosed using the World Health Organization (WHO) 2016 criteria for MPN [2]. They share common mutations; the vast majority of patients with PV, ET, and PMF have a mutation in either the JAK2, CALR, or MPL genes [3]. Patients with Philadelphia-negative MPN are at risk of developing complications including thrombosis, bleeding, leukemic transformation, or a fibrotic phase of the disease. A substantial number of patients are in the reproductive age groups, and patients are diagnosed earlier, and with treatment, patients have improved life expectancy. As a result, the fertility of patients with these MPN became an important upcoming concern. Fertility is affected by several factors, including age, significant systemic disease in either partner, and exposure to environmental toxins, medications, or radiation [4]. The disease effect and the effect of the received treatment on male fertility are essential questions to be addressed. For example, hydroxycarbamide is used to treat PV; in males with sickle cell anemia, treatment with hydroxycarbamide had significant changes in the semen parameters [5]. The treatment effect of male fertility in Philadelphia-negative MPN is not well studied. Several studies have looked at the fertility of female CML patients, but there are a limited number of studies worldwide that addressed the outcome of male patients with Philadelphia-negative MPN. This study is designated to assess the fatherhood of Philadelphia-negative MPN male patients in chronic reproductive and biological functions and the well-being of their offspring.

## Materials and methods

Methodology

This is a mixed-design pilot study that was conducted by phone interviews with Philadelphia-negative MPN male patients being followed up at National Center for Cancer Care and Research (NCCCR) to evaluate male fertility in the patients, including both on current and past treatment. Patients were interviewed regarding fertility-related information such as the number of children they had while on treatment, their way of delivery, and their offspring’s health status. All included patients (Table 1) were followed up in our center between January 1, 2008, and January 1, 2020. Patients aged more than 18 years old and agreed to be interviewed over a phone call.

**Table 1:** Inclusion and exclusion criteria of Philadelphia-negative MPN patients MPN: myeloproliferative neoplasm, PV: polycythemia vera, ET: essential thrombocythemia, PMF: primary myelofibrosis, WHO: World Health Organization

Inclusion criteria
Adult male patients aged ≥18 years old diagnosed with Philadelphia-negative MPN (ET, PV, MF, PMF, and prefibrotic PMF) according to the 2008 and/or 2016 WHO criteria and actively receiving treatment including hydroxycarbamide, pegylated interferon 2 alpha (PEG-IFNA2a), and ruxolitinib
Patients with no previous fertility problems, e.g., no primary infertility or erectile dysfunction or priapism
Exclusion criteria
Patients with Philadelphia-positive MPN (chronic myeloid leukemia)
Patients known to have infertility before the diagnosis of Philadelphia-negative MPN
Patients with infertility after the diagnosis of Philadelphia-negative MPN (ET, PV, MF, and PMF)
Patients with a clear underlying cause of infertility, e.g., vasectomy
Female partners with documentation by a gynecologist for infertility (any mother-related cause, whether endogenous or exogenous)

Ethical approval

The study was reviewed and approved by the Medical Research Center (approval number MRC-01-20-812).

Participants

This study included adult male patients aged ≥18 years old diagnosed with Philadelphia-negative MPN (ET, PV, MF, PMF, and prefibrotic PMF) according to the 2008 and/or 2016 WHO classification. All patients are residing in the state of Qatar, with regular follow-up in NCCCR.

Study population and study setting/location

The study reviewed 124 patients by phone interview and records from the National Center for Cancer Care and Research (NCCCR) in Doha, Qatar.

## Results

**Figure 1:**
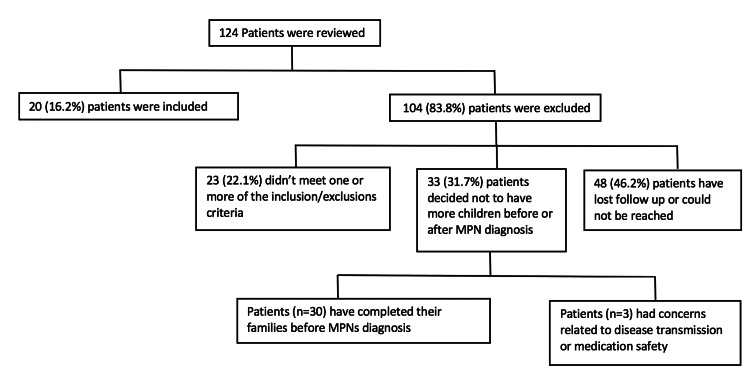
The number of patients included and excluded from the study

Of the 124 patients interviewed, only 20 (16.2%) patients had met the inclusion criteria (Figure 1). The majority of the patients were lost to follow-up or could not be contacted, and 28.8% of the patients had their families completed by the time of diagnosis. Only a few patients had completed their families due to concern regarding the medication or the disease’s effect on the offspring (n=3), and many patients decided to stop their families after the diagnosis of MPN (n=30). The treatment received included hydroxycarbamide (n=8), pegylated interferon 2 alpha (n=10), ruxolitinib (n=1), and phlebotomy (n=1). The mode of delivery was normal vaginal delivery in 71.4% (n=20) of the pregnancies. The total number of offspring was 30, and the total number of conceptions was 30, with two twin pregnancies. Three stillbirths were reported, one intrauterine fetal death, and one baby of a twin died in utero (Table 2). The mean age of children born after the diagnosis was 6.1 years, ranging between one month and 21 years.

**Table 2:** Patient characteristics

Characteristics	Number
Age at diagnosis	Mean age: 34 years; range: 18-48 years
Number of patients	
Essential thrombocythemia	n=11
Polycythemia vera	n=7
Primary myelofibrosis	n=2
Prefibrotic primary myelofibrosis	n=0
Treatment received	
Hydroxycarbamide	n=8
Pegylated interferon 2 alpha	n=10
Ruxolitinib	n=1
Phlebotomy	n=1
Duration of treatment	Mean: 8.5 years; range: 1-25 years
Duration of treatment before conception	Mean: 4.7 years; range: 16-1 years
Total number of conceptions	n=30 (two twin pregnancies)
Total number of offspring	n=30
Number of abortions	n=2
Mode of delivery	n=28 pregnancies
Normal vaginal delivery	n=20
Cesarian section	n=8
Stillbirth or intrauterine birth demise	n=2 (one was a twin who died in utero)
Age of children born after MPNs diagnosis	Mean: 6.1 years; median: 6 years; range: 1 month-21 years

## Discussion

Infertility is defined as the inability of a couple to conceive after 12 months of regular intercourse without contraception in women under 35 years and after six months of regular intercourse without contraception in women 35 years or older [6]. Worldwide, it is estimated that around 1.9% of women aged 20-44 who wished to start a family were unable to have their first child and that 10.5% of women who already had a live birth were unable to have a second child [7]. Infertility is higher in Eastern Europe, North Africa, the Middle East, and Sub-Saharan Africa [7]. In cancer survivors, reduced fertility can be related to cytotoxic medications damaging the reproductive organs directly or by altering the hypothalamic-pituitary-gonadal axis [8]. Additionally, fertility in patients with MPN might be caused by radiation therapy, surgery, the disease process itself, or priapism, as in ET or chronic myeloid leukemia [8,9]. Such an effect on fertility can be temporary or permanent.

When it comes to fertility in cancer survivors, there are major points to be considered. First, infertility itself is associated with a significant impact on couples; it is associated with profound emotional, psychological, and cognitive effects on patients [10]. Searching the literature, we found that the psychological impact of infertility is more described in females; however, many studies showed the impact on both sexes. Psychiatric disorders were found in 69.6% of female patients referred from fertility clinics [11]. Additionally, Wichman et al. found the rate of anxiety to be prevalent in both sexes, with 50.3% of men and 66% of women attending in vitro fertilization [12]. Second, cancer itself produces huge psychiatric morbidity in this group of patients; a meta-analysis described this finding in young adult cancer survivors. The most commonly identified psychiatric disorders were mood disorders (odds ratio (OR): 1.36; 95% CI: 1.19-1.55), followed by anxiety disorders (OR: 1.16; 95% CI: 1.05-1.28), and these disorders were significantly reported in females [13]. From the mentioned studies [12,13], it is apparent that the impact of fertility in cancer survivors is high, with slightly more impact on females; however, the impact in males may be underestimated or not well studied as fewer studies looked at that and the close percentage between the two sexes. Third, patients with cancer face concerns about their survival, response to treatment, quality of life, and additional concerns about fertility, which can be compromised. Such matters include the risk of having a healthy child and worries regarding the development of similar malignancy in their offspring. Such complex and challenging questions will probably make counseling of such patients convoluted, and these patients may refrain from having children, which complicates their sexual life.

Fortunately, there are some answers to these concerns regarding the risk of congenital and chromosomal abnormalities, miscarriage, stillbirth, and the occurrence of malignancy in their offspring. Several studies have shown that cancer survivors’ offspring are not at a higher risk of having chromosomal abnormalities [14]. With the exception of patients with hereditary or familial cancer syndrome, the risk of having cancer in the offspring was not significantly higher, as reported in many international studies that had a good follow-up [15,16]. Our data show similar results, as there are no reports of similar malignancy in our patients’ offspring. A significant percentage of patients diagnosed with Philadelphia-negative MPN are young; the mean age of patients meeting the inclusion criteria at the time of diagnosis was 34 years. This younger age at the time of diagnosis is probably affected by the fact that the population of the studied country is young, with the majority of them in their productive years. This means that a large number of patients in the reproductive age group are sexually active. Having a diagnosis of MPN will put these patients in substantial psychological distress due to the unknown outcome about their fertility and sexual life and the fear of possible consequences on their children. Fertility in patients with Philadelphia-negative MPN is mainly studied in female patients, and meta-analysis showed that they had a lower fertility rate than the general population; the risk is higher for PV compared to ET and lowers with aspirin use [17]. With such an effect of reduced fertility in females, it is crucial to know the impact of MPN on fatherhood.

The most extensive database on male cancer survivor fertility comes from the Childhood Cancer Survivor Study (CCSS), which showed that males who were not surgically sterile were less likely to sire a pregnancy compared to siblings without cancer (hazard ratio (HR): 0.56; 95% CI: -0.49-0.63) [18]. Such a risk is more pronounced in patients who had been exposed to a radiation dose of >7.5 Gy to the testes, a higher cumulative alkylating agent dose score, and those treated with cyclophosphamide or procarbazine. However, patients in this large cohort were primarily with leukemia, lymphoma, and solid organ malignancies such as bone tumors and sarcomas; no MPN patients were mentioned in the study. Most MPN patients do not routinely receive radiation therapy or testicular radiation, and the type of treatment received in Philadelphia-negative MPN is usually different; no alkylating agents are used. As a result, the effect of treatment on patients with MPN could vary completely from those in the CCSS.

The treatment used in MPN includes hydroxycarbamide, which is well studied in patients with sickle cell disease; it has been used to treat sickle cell disease for more than 30 years. Studies showed that hydroxycarbamide is associated with altered sperm parameters, and these changes can be reversible or irreversible with stopping the medication [13,19]. There are several factors contributing to infertility in sickle cell patients; however, some reviews suggest that hydroxycarbamide contributes to infertility in male patients with sickle cell disease [14,20]. Moreover, hydroxycarbamide is associated with an increased risk of second malignancy compared to PEG-IFNA2a [18,21]. The effect of pegylated interferon 2 alpha on male fertility is studied in patients with chronic hepatitis C infection without cirrhosis [22]. The study involved only 40 male patients. It showed that after treatment with pegylated interferon 2 alpha, patients had a significant change in the level of follicle-stimulating hormone and luteinizing hormone, decreased testosterone level, and reduced sperm count and motility; it has negative effects on sperm count [22]. Data regarding ruxolitinib’s effect on sperm count is lacking, but the effect of tyrosine kinase inhibitors such as imatinib shows that imatinib can cross the testis’ blood barrier and might have an effect on sperm count and motility [23]. In patients with CML, dasatinib and nilotinib have a significant effect on semen parameters; however, there is no evidence of the expected effect of ruxolitinib on male fertility [24]. Another medication used in MPN treatment is aspirin. Aspirin was found to affect semen quality; several human and animal studies showed that aspirin can decrease the formation of testicular prostaglandins, reduce motility, reduce viability, lower semen volume, lower sperm concentration, and reduce nitric oxide, which might increase oxidative injury to sperm [25]. However, the clinical significance and degree of the effect on male fertility are difficult to estimate as many patients are using aspirin for different categories of diseases without reporting a significant impact on their fertility.

One of the modalities for the treatment of polycythemia vera is phlebotomy. There are no clear studies about the effect of phlebotomy on male fertility. However, blood transfusion in patients with sickle cell disease is associated with a significant effect on libido, increase in sperm parameters, and activity of the hypothalamic-pituitary axis [26]. The reverse is not always true, and it is difficult to conclude that phlebotomy will decrease the gonadal hormone activity as phlebotomy brings hemoglobin back to the normal range in polycythemia vera, and if untreated, iron overload can cause suppression of the pituitary-gonadal axis.

Another factor that can affect fertility is the disease itself, as cancer is associated with raised inflammatory cytokines, which are raised in other systemic illnesses and can affect the hypothalamic-pituitary axis. In both acute and chronic illnesses, the gonadal axis can be suppressed, resulting in gonadal hypofunction [27,28]. However, there is no proof that a similar process happens with MPN. To the best of our knowledge, there are no previous studies that support such findings. However, the result of our study showed that these patients are fertile despite the limitation of the number of the involved participants. The majority of the patients were lost to follow-up, making the sample size small, and hence, the result has many limitations. Our data showed that only two patients had lost their pregnancy in their first trimester; compared to the general population, this ratio is not high as abortion is seen in up to 30% of normal pregnancies [29]. The data showed that patients with MPN had an average of 4.22 years of treatment before having their children, which is a significant duration if there is any effect of medication on the patients’ fertility to occur. The average children’s age was 7.53 years, and until this age, there were no reports of MPN-related disease in the children, although a longer duration of follow-up is needed to address the occurrence of MPN in the second generation.

Apart from sexual function and employability, feeling well enough to be a good parent is one of the most significant emotional well-being indicators in cancer survivors [30]. It is crucial to discuss the goal of therapy with patients as treatment may result in jeopardized fertility, which may affect the patients’ preference of treatment. However, only a few patients showed genuine concern regarding the effect of the disease on their offspring and decided that they would not try to conceive (n=3); such a number was smaller than expected. The low concern may be because many physicians do not disclose the fact that MPNs are true malignancies to their patients, and patients do not deal with their diseases like cancer.

## Conclusions

This pilot study showed that most Philadelphia-negative MPN male patients on treatment had their offspring born normally with no delivery complications, no reported congenital anomaly or growth retardation, and no report of MPN-related cancers. The main limitation of the study is the small number of included patients. Further studies with a larger sample size are required to fully understand the effect of medications on the outcome of fatherhood in Philadelphia-negative MPN patients. Nonetheless, there is a call for attention for better education to patients, addressing the possible psychological fear and concerns of having an unsatisfactory effect on their fertility/offspring, targeting better acceptance and adherence to treatment.
